# A Workflow for Patient-Individualized Virtual Angiogram Generation Based on CFD Simulation

**DOI:** 10.1155/2012/306765

**Published:** 2012-11-04

**Authors:** Jürgen Endres, Markus Kowarschik, Thomas Redel, Puneet Sharma, Viorel Mihalef, Joachim Hornegger, Arnd Dörfler

**Affiliations:** ^1^Pattern Recognition Lab, Department of Computer Science, Friedrich-Alexander University of Erlangen-Nuremberg, Martensstrasse 3, 91058 Erlangen, Germany; ^2^Angiography & Interventional X-Ray Systems, Healthcare Sector, Siemens AG, Siemensstrasse 1, 91301 Forchheim, Germany; ^3^Corporate Research and Technology, Siemens Corporation, 755 College Road East, Princeton, NJ 08540, USA; ^4^Erlangen Graduate School in Advanced Optical Technologies (SAOT), Friedrich-Alexander University of Erlangen-Nuremberg, 91052 Erlangen, Germany; ^5^Department of Neuroradiology, Friedrich-Alexander University of Erlangen-Nuremberg, Schwabachanlage 6, 91054 Erlangen, Germany

## Abstract

Increasing interest is drawn on hemodynamic parameters for classifying the risk of rupture as well as treatment planning of cerebral aneurysms. A proposed method to obtain quantities such as wall shear stress, pressure, and blood flow velocity is to numerically simulate the blood flow using computational fluid dynamics (CFD) methods. For the validation of those calculated quantities, virtually generated angiograms, based on the CFD results, are increasingly used for a subsequent comparison with real, acquired angiograms. For the generation of virtual angiograms, several patient-specific parameters have to be incorporated to obtain virtual angiograms which match the acquired angiograms as best as possible. For this purpose, a workflow is presented and demonstrated involving multiple phantom and patient cases.

## 1. Introduction

Cerebrovascular diseases are beneath cardiovascular diseases the leading cause of death among industrialized countries [1]. One clinical pathology concerning the cerebrovascular system is intracranial aneurysms, abnormal bulges within the vasculature. According to the study in [2], the prevalence of unruptured intracranial aneurysms in the general population is estimated to be up to 5%. Aneurysms threaten the patients' health in case of rupture, which will lead to a subarachnoid hemorrhage (SAH) and hence may cause a hemorrhagic stroke with severe clinical consequences. For the case of intracranial aneurysms, 30% of all patients will die within the next 30 days, 30% will develop disabilities, and only the remaining part will almost completely recover [3, 4]. However, most of the aneurysms will never rupture. For example, out of more than 10–12 million people in the US which are estimated to have an intracranial aneurysm, about 27,000 cases per year will suffer from subarachnoid hemorrhage caused by rupture events [3].

In the management of unruptured intracranial aneurysms, different preventive treatment options are established. In a neurosurgical procedure, a metal clip is placed during an open surgery along the neck of the aneurysm to prevent blood from flowing into the aneurysm dome and hence disable the possibility of rupture [5]. In an endovascular treatment, small coils are placed within the aneurysm dome. The intention of those coils is to reduce the blood flow inside the aneurysm, leading to thrombosis and finally to an occlusion of the aneurysm. A recent interventional approach is based on the placement of flow diverting devices within the parent artery, which also aims at reducing blood flow inside the aneurysm [5].

For endovascular treatment, X-ray angiography [6] is mandatory to visualize the aneurysm as well as parent vasculature. By intraarterially injecting contrast agent, vessel structures can be visualized in addition to catheter devices. Modern systems, where source and detector are mounted at both ends of a movable, C-shaped fixture (C-arm), are capable of acquiring 2D digital subtraction angiography (DSA) images at high frame rates, which allows to observe the distribution of injected contrast agent over time. Additional plane DSA sequences unveil a lot of flow dynamic information about the hemodynamic behaviour. In addition, by rotating the C-arm around the object, static volumes can be reconstructed in a CT-like fashion (3D rotational angiography, 3D RA) [7].

However, since all treatment options imply risks for the patients, reliable parameters for aneurysm risk classification, treatment planning, and assessment are needed. Besides geometric properties of the aneurysm itself, an increasing interest is shown for hemodynamic parameters such as pressure, wall shear stress, and blood flow velocity. Due to insufficient methods of measuring those quantities *in vivo*, computational methods—that is, numerical simulations—are investigated in order to obtain those quantities, as, for example, given in [8, 9].

However, a reliable validation of the simulation results is required prior to applications in clinical environments, for which Ford et al. [10] suggested the generation of virtual/synthetic angiograms based on CFD simulation results, and a succeeding comparison of virtual and the corresponding real angiograms. One major aspect concerning this validation method for CFD simulation results is the definition of patient-specific boundary conditions. Since those patient-specific parameters are generally not available for acquired 2D DSA sequences, *in vitro* studies based on cerebral aneurysm phantoms have been performed, where parameters such as blood flow velocities at vessels proximal to the aneurysm are known [11, 12].

Furthermore, in certain DSA acquisitions, the injection of the contrast agent is done manually which leads to variations in the injection profile as well as in the timing with respect to the patient's heart phase. Using standardized injection profiles for virtual angiography will then lead to deviations of the virtual angiogram when compared to the real one.

Beneath the aspect of validation, further studies have been published using the virtual angiography technique; for example, for visualizing outcomes of virtual treatment techniques [13, 14] or for evaluating the outcome of CFD simulation results [15–17]. Clinical applications may benefit from virtual angiograms in a way that these image sequences can be generated without the use of applying further X-ray radiation dose and injecting additional contrast agent, and they can be generated for arbitrary angulations, independent of mechanical limitations such as unreachable C-arm angulations. As a visionary future aspect—not taking into consideration the need for a validation of CFD approaches—virtual angiograms may eventually completely replace real angiograms, such that only a 3D RA is acquired for diagnostics, whereas all dynamic information is entirely based on CFD simulation and virtual angiogram generation. Finally, virtual angiography represents a familiar way for illustrating CFD simulation results, which would be hard to interpret otherwise.

In this paper, we extend the methods proposed in [10] by further incorporating patient- and treatment-specific parameters to obtain virtual angiograms aimed at matching the corresponding real angiograms as accurately as possible. Our basic workflow has already been published in [18]. The contributions of this extended work are an additional synchronization of the heart state at the beginning of virtual and acquired DSA sequences as well as a more detailed examination of the accuracy of the results with respect to quantitative error measurements.

Our paper is structured as follows. In Section 2, the basics of the underlying CFD computations are presented, the mathematical model of virtual contrast agent (contrast medium) injection and propagation is demonstrated, and methods for extracting several patient-individual parameters are covered. Furthermore, Section 2 details the generation of the virtual angiograms as well as our approaches towards the quantitative comparison of virtual and real angiograms. These methods are embedded in a workflow which is then applied to both phantom and patient cases. In Section 3, results are presented and discussed. We finally draw our conclusions in Section 4.

## 2. Materials and Methods

**Figure 1:**
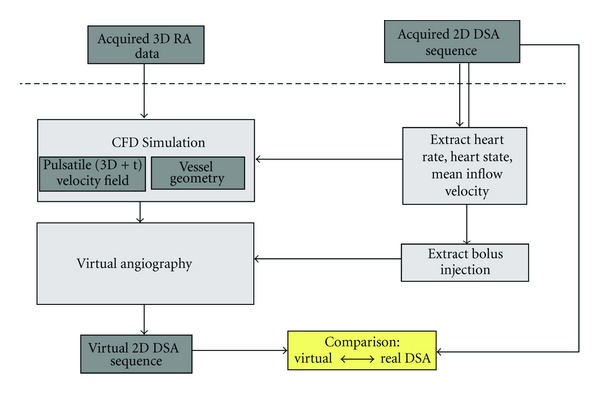
Virtual angiography workflow.

Figure 1 provides a schematic overview of the distinct steps for generating virtual angiograms. For these methods, two different types of imaging data are used. On one hand, a volumetric 3D RA image provides geometric information for both the CFD simulation and the virtual angiography. On the other hand, a 2D DSA sequence—ideally acquired at a high frame rate (e.g., 30 frames per second or higher)—will serve as input data for a patient-specific parameter extraction and, afterwards, as ground truth for comparison. As a result, virtual (synthetic) 2D DSA sequences from arbitrary viewing directions are generated.

First, patient-specific information concerning the heart rate and heart state will be extracted from the 2D DSA sequence. This information will then be used for adapting CFD simulation parameters. Second, as additional patient-specific information, the contrast bolus injection profile is extracted from the acquired angiogram. An individualized virtual angiogram is subsequently created based on the CFD output. Finally, the resulting virtual angiogram is compared both qualitatively and quantitatively with the real angiogram.

This approach represents an essential step towards the validation of the CFD results. If the virtual angiogram matches the real angiogram closely, the user may become confident of the application of CFD methods and hence generate and evaluate further virtual angiograms from additional viewing directions without applying additional X-ray dose to the patient and without injecting further contrast medium. This means that, eventually, the computation of further virtual angiograms might replace the acquisition of further real angiograms. Note that, using virtual angiography, even viewing directions are possible that cannot be reached by the C-arm due to mechanical limitations (e.g., due to patient/table collision).

### 2.1. CFD Simulation—Hemodynamic Simulation of Cerebral Blood Flow

**Figure 2:**
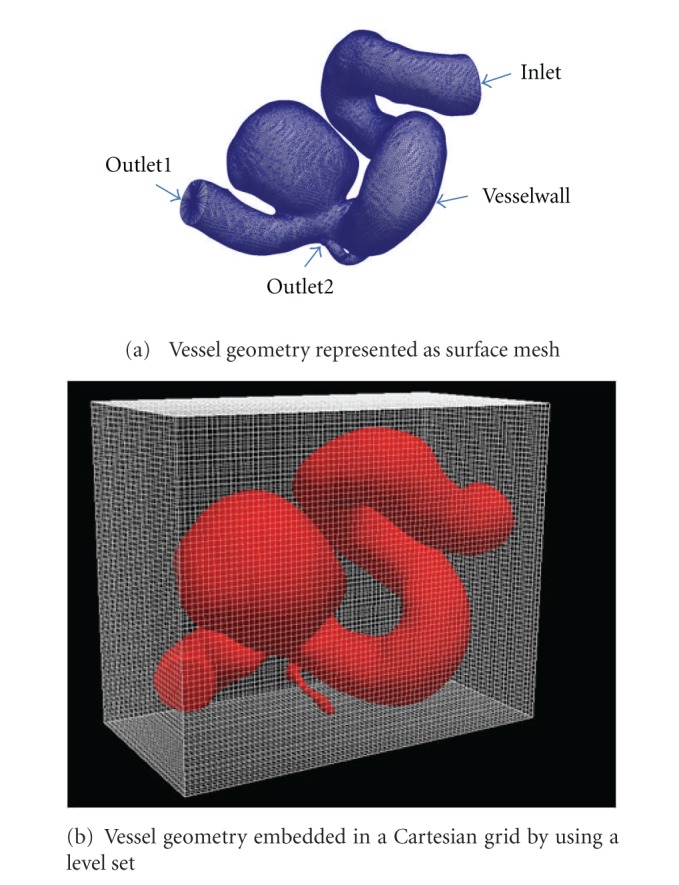
Vessel geometry for CFD simulation.

For the computation of the flow in the cerebral vessels, the blood is modeled as a Newtonian fluid with prespecified density (*ρ* = 1050  kg/m^3^) and viscosity (*μ* = 0.004  Pa · s). The basic principles of conservation of mass and momentum are applied by numerically solving the Navier-Stokes equations under appropriate boundary conditions. Under our simulation framework, the complex vessel geometry, as shown in Figure 2(a), which is provided as a surface mesh, is embedded in a Cartesian grid by using a level set, compare Figure 2(b) [19]. This provides an automatic domain setup and allows the user to bypass the time-consuming step of mesh generation [20].

After computing a level set *φ* with positive values inside the vessel, we solve the Navier-Stokes equations

(1) $$\begin{array}{c} \rho \left(\varphi \right)\left(\frac{\partial u}{\partial t}+u\cdot \nabla u\right)=-\nabla p+\mu \left(\varphi \right)\Delta u+F,\\ \nabla \cdot u=0,\\ \rho \left(\varphi \right)=\rho^{1}\left(\varphi \right)H\left(\varphi \right)+\rho^{2}\left(\varphi \right)\left(1-H\left(\varphi \right)\right),\\ \mu \left(\varphi \right)=\mu^{1}\left(\varphi \right)H\left(\varphi \right)+\mu^{2}\left(\varphi \right)\left(1-H\left(\varphi \right)\right),\\ H\left(\varphi \right)=\{\begin{array}{cc} 1, & \varphi >0,\\ 0, & \varphi <0. \end{array} \end{array}$$

The Heaviside function *H* distinguishes sharply between the solid and the fluid components of the domain, while we use second-order accurate spatial extrapolation across the boundary when imposing boundary conditions. The equations are discretized and solved iteratively for velocity and pressure. We use a fractional step method [21] that computes in a first step an intermediate velocity field using the nonlinear advection-diffusion equation for velocity and then projects the intermediate velocity onto the field of divergence-free and tangent to the vessel boundary vector fields.

For the velocity advection, we use a second-order upwind Van-Leer slope limiting method, while for the diffusion force components, we use a semi-implicit approach as in [22] which is second-order accurate in space and unconditionally stable in 3D. The pressure Poisson equation (PPE) is solved using a multigrid preconditioned conjugate gradient solver. After the PPE is solved and the updated pressure field is determined, the fluid domain velocity is updated by subtracting the pressure gradient. The body force field *F* in (1) can be used to include forces due to flow diverter embedded geometries, as we proposed in [23].

For applying the boundary conditions, the inlet is completely embedded inside the Cartesian grid, and Dirichlet boundary conditions for velocity are enforced using linear extrapolation from the interior of the domain using an extrapolation routine adapted from [24]. A time-varying velocity field is applied at the inlet, which is modeled spatially as a plug profile. The outlets are modeled with constant pressure boundary conditions. The computations are performed using time steps constrained by the CFL condition [25], while the spatial resolution was in the range of 5 · 10^5^ cells, chosen such that the velocity differs less than 1% when compared to the refined grid.

### 2.2. Virtual Angiography—Simulated Transport of Contrast Agent and Its Visualization

In our approach, contrast agent passing through the vascular territory under consideration is modeled as a set of *n* discrete particles

(2) $$\begin{array}{c} \Omega =\left\{\rho_{i}\right\},\;\;\;\rho_{i}\in {\mathbb{R}}^{3},\;i\in 1,\ldots ,n,\;n\in \mathbb{N}. \end{array}$$

The particles are assumed to be both mass- and dimensionless; hence, there is no interaction between particles (e.g., there are no (in)elastic collisions). Each particle **ρ**_*i*_ is defined by its location in ℝ^3^ and is freely movable within space which means that its position is not restricted to grid points. Note that other approaches towards the generation of virtual angiograms are based on the numerical solution of an advection-diffusion equation in order to simulate the transport of contrast agent, see [10], for example. Our particle-based method can be seen as a straightforward alternative to a scheme that explicitly models the physics of contrast medium propagation using a partial differential equation [26]. By using this discrete scheme, additional analysis based on the particle representation can be included for flow quantification; for example, particle residence times [27] or further visualization techniques such as streamlines, streaklines, or pathlines may be employed.

**Figure 3:**
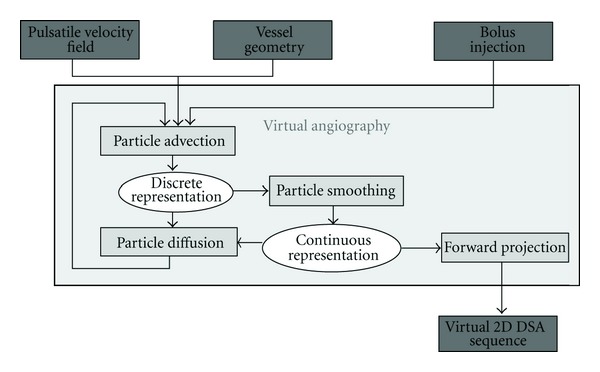
Virtual angiography algorithm.

Two distinct physical processes are involved in the transport of contrast medium through the vasculature. On one hand, an advective process propagates contrast agent based on an underlying velocity field, which is generated by the CFD solver. On the other hand, a diffusive process causes the contrast agent to mix autonomously with blood, which leads to a homogenization of both substances.  Figure 3 illustrates the algorithm for performing the simulated transport of contrast agent consisting of advection, diffusion, and an additional smoothing procedure, which is used to transform the discrete particle set *Ω* into a corresponding continuous representation. In each time step, the particle set is processed sequentially. First, advection is applied to each particle. The resulting particle set is then transformed into a continuous representation (particle smoothing), from which a concentration gradient field is then obtained. Finally, the particle set is processed again according to the calculated gradient (particle diffusion).

**Advection**

Taking into consideration only the advective part of the transport process, the trajectory of a single particle **ρ**_*i*_ can be characterized independently from all other particles. This trajectory can be described as the solution of the ordinary differential equation (ODE)

(3) $$\begin{array}{c} {\dot{\rho }}_{i}\left(t\right)=f\left(t,\rho_{i}\left(t\right)\right), \end{array}$$

where **ρ**_*i*_ denotes the spatial position of the particle and *t* represents the time. For a unique solution, an initial value

(4) $$\begin{array}{c} \rho_{i}\left(t_{i}=0\right)=\rho_{i,0} \end{array}$$

has to be specified. This value corresponds to the point in space and time where the particle gets injected into the vasculature.

In (3), *f* : ℝ × ℝ^3^ → ℝ^3^ denotes the function representing the time- and space-dependent velocity field. The function *f* itself is unknown; only the function values—representing the velocities at the nodes of the computational grid—are computed by the underlying CFD solver. Hence, this equation is not solvable analytically and, consequently, a numerical solution has to be considered, for which an explicit fourth-order Runge-Kutta scheme, given by

(5) $$\begin{array}{c} \rho_{i}\left(t+\delta t\right)=\rho_{i}\left(t\right)+\frac{1}{6}\cdot \left(k_{1}+2k_{2}+2k_{3}+k_{4}\right),\\ \text{where}\;\;k_{1}=\delta t\cdot f\left(t,\rho_{i}\left(t\right)\right),\\ k_{2}=\delta t\cdot f\left(t+\frac{1}{2}\delta t,\rho_{i}\left(t\right)+\frac{1}{2}k_{1}\right),\\ k_{3}=\delta t\cdot f\left(t+\frac{1}{2}\delta t,\rho_{i}\left(t\right)+\frac{1}{2}k_{2}\right),\\ k_{4}=\delta t\cdot f\left(t+\delta t,\rho_{i}\left(t\right)+k_{3}\right), \end{array}$$

is used [26].

For the choice of *δt*, the CFL condition [25], which correlates the time step, the given flow velocities, and the resolution of the underlying computational grid, is taken as a reference.

Due to the discretization in time, the particles may be advected such that they leave the vessel through the boundary, which corresponds to a flux of contrast agent through a vascular wall. To prevent this, these particles will be kept inside by bouncing them at the vascular wall back into the vessel. This represents a physically reasonable approach under the assumption of rigid vascular walls.

**Diffusion**

For simulating the diffusive process according to Fick's law [28], given by

(6) $$\begin{array}{c} v_{\text{Diff}}\left(x,t\right)=-D\cdot \frac{\partial C\left(x,t\right)}{\partial x}, \end{array}$$

the discrete particle set *Ω* is transformed into a continuous representation *C*(**x**, *t*) describing the concentration of contrast agent (see Smoothing). According to (6), the direction and the magnitude of the diffusive movement **v**_Diff_ is obtained by calculating the spatial gradient of the concentration image *C*(**x**, *t*), scaled by a substance-dependent diffusivity coefficient *D*. The resulting gradient image is subsequently used as the velocity image for advancing the particles according to diffusion.

Since contrast agent is restricted to the interior of the vessels, high concentration differences will occur at vessel boundaries, which in turn will generate large contrast medium concentration gradients. Consequently, contrast agent (i.e., particles) touching the boundary will keep on diffusing strongly towards the boundary, which results in those particles being bounced back into the vessel. Therefore, an intermediate step is taken. After the discrete-continuous transformation, zero gradients are assured at the vessel boundary by extending the concentration from inside the vessel over the boundaries. This is achieved through the use of a distance transform [29], where each voxel outside the vessel is assigned an additional vector pointing to the closest voxel inside the vessel. This vector is then used to copy the concentration values from voxels inside the vessel to corresponding voxels outside the vessel.

**Smoothing**

For the reprojection, that is, the forward projection, of contrast agent concentration volumes as well as for the simulated diffusion process (in particular, for the calculation of the gradient vector field), the discrete particle representation is required to be transformed into a continuous representation of contrast agent such that its distribution is available on a regular grid. This is achieved by the following smoothing step:

(7) $$\begin{array}{c} C_{\text{discrete}}=\delta \left(x\right)\to C_{\text{continuous}}=\sum_{\rho_{i}\in \Omega }f\left(x,\rho_{i}\right), \end{array}$$

where

(8) $$\begin{array}{c} \delta \left(x\right)=\{\begin{array}{cc} 1, & \text{if}\;\;x-\rho_{i}=0,\;\;\rho_{i}\in \Omega ,\;\;x\in {\mathbb{R}}^{3},\\ 0, & \text{else}. \end{array} \end{array}$$

This transformation describes a smearing (or smoothing) operation of a particle over its spatial neighborhood. The range and the way the particle gets smoothed is thereby specified by the function *f*(**x**), for which a Gaussian distribution, given by

(9) f(x,ρi)=1(2π)3|Σ|e−(1/2)(x−ρi)TΣ−1(x−ρi),        ρi∈Ω,  x∈ℝ3,

is chosen. A smoothing parameter *σ* is thereby used for the covariance matrix Σ = *σ* · *I*_3_, where *I*_3_ denotes the identity matrix, in order to parametrize the amount of smoothing. As the mean value for the Gaussian distribution, the particle position **ρ**_*i*_ is used.

**Figure 4:**
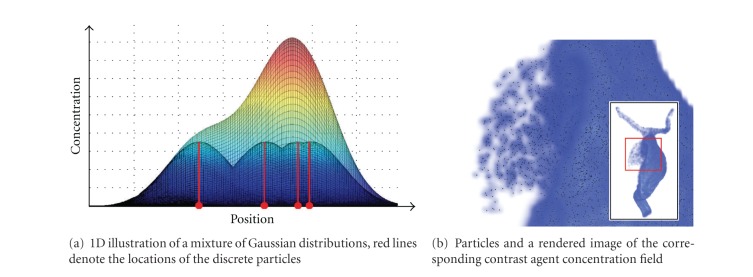
Particle smoothing for the sake of reprojection and the determination of contrast agent gradients.

Taking all particles into consideration, the final continuous distribution is hence a mixture of single density distributions (see Figure 4). This mixture density can then be sampled on the desired grid, which is here chosen in correspondence to the grid from the CFD solver (i.e., number of grid points per dimension and grid spacing) to keep the properties of the vessel geometry.

Using a Gaussian distribution as smoothing function has several benefits. First, the exact position of the particle between grid points is respected (by using it as the mean value of the Gaussian distribution); other solutions, such as assigning the particle to the nearest grid point, would typically shift the original particle position. Second, the Gaussian function allows to model a spatially symmetric smoothing. Third, when varying the smoothing parameter *σ*, the integral of the Gaussian and hence the total amount of spatially distributed contrast agent remains constant.

### 2.3. Patient-Specific Parameter Extraction

**Heart Rate**

The heart rate of each patient varies dependent on the patient's age, physical constitution, and so forth. For assuring a synchronized pulsatile pattern of real and virtual angiograms, the use of an average heart rate taken from the medical literature should thus be avoided. Measuring the patient-specific heart rate before treatment is also disadvantageous, because differences may occur between the resting heart rate and the heart rate during the treatment due to physiological factors such as stress or medication. Hence, it is desirable to have this information for the exact time when the patient is being treated.

**Figure 5:**
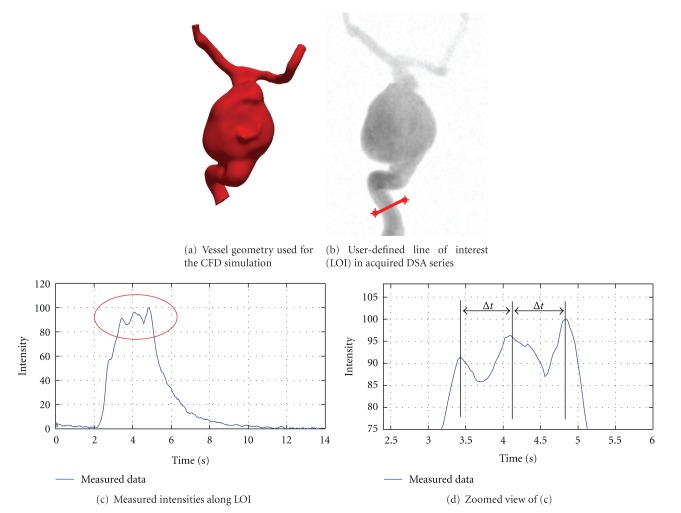
Extraction of heart rate information. The location of the line of interest (LOI), (b), corresponds to the inlet of the geometric model for the CFD simulation (a). Based on the measured intensities (c), the time between subsequent intensity peaks (d) is used for estimating the patient's heart rate.

To obtain the patient-specific heart rate, a user-defined line of interest (LOI) within the acquired 2D DSA series (Figure 5) is used. For each image of the 2D DSA series, the image intensities are integrated along this LOI. This results in a time-intensity curve which characterizes the concentration of contrast agent over time. Succeeding intensity peaks of the measured data are then identified, out of which the heart rate *h* is calculated using

(10) $$\begin{array}{c} h=\frac{1}{n-1}\sum_{i=2}^{n}\left(p_{i}-p_{i-1}\right), \end{array}$$

where *p*_*i*_ denotes the time point of the *i*th intensity peak, and *n* represents the total number of identified intensity peaks within the DSA series (Figure 5).

This heart rate is then used for the CFD simulation; the inflow velocity profile is adapted according to the calculated duration.

**Heart State**

In order to generate virtual angiograms that match the corresponding real angiograms as accurately as possible, not only the duration of a cardiac cycle, but also the state of the heart at the beginning of both angiograms, which affects the blood flow velocities and hence the propagation of contrast agent, must be synchronized.

**Figure 6:**
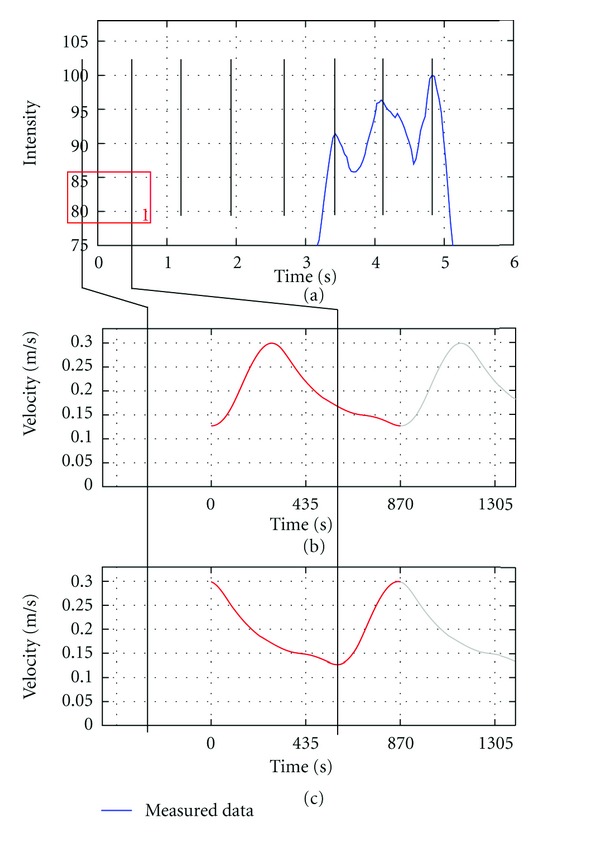
Heart-state synchronization. In (a), black vertical lines denote the extrapolated intensity peaks. In (b), the standard inflow velocity profile (red) before adaptation is shown in combination with the intensity peaks. Assuming a correspondence of low blood velocities and high opacification, the inflow velocity profile does not match to the extrapolated intensity peaks. In (c), the adapted (shifted) inflow velocity profile is shown which is then used for the CFD simulation.

For this purpose, the time-intensity curve based on the proposed LOI (Figure 5(b)) is used and the intensity peaks are identified. Under the assumption of a periodic heart beat, these peaks are extrapolated backward in time to the beginning of the time-intensity curve (Figure 6(a)).

Now, the positions of the extrapolated intensity peaks next to the beginning of the acquired DSA signal are observed (Figure 6(a), box 1). Based on these positions, the CFD inflow velocity profile, which has already been adapted for the patient-specific heart rate, is periodically shifted such that this velocity profile would produce the same pulsatility pattern and hence the same intensity peaks as acquired for the patient. The relation between intensity peaks and inflow velocity profile is given by a correspondence of high velocities and low contrast agent intensities, since high velocities cause a large amount of blood to pass the injection point per time, but still absorbing the same amount of contrast agent (assuming a constant injection rate). This leads to a lowered contrast agent concentration and thus to reduced intensities on the acquired DSAs. By shifting the inflow profile, low velocities are assured to match the intensity peaks (Figure 6(c)).

**Inflow Velocities**

Inflow velocities are estimated by subsequently performing CFD simulations with varying mean inflow velocity. For each CFD simulation, a virtual angiogram is generated, and time-intensity curves are acquired at certain regions of interest for the real and virtual angiogram; for example, proximal to the aneurysm. Based on a comparison of these time-intensity curves, the mean inflow velocity is optimized manually to match proximal flow patterns.

**Bolus Injection Profile**

In angiographic procedures, injecting contrast agent is either performed using a mechanical injection regulator or an injection by hand. Especially for manual injections, the profile (e.g., duration, pressure) at which a certain amount of contrast agent is inserted into the respective artery differs from injection to injection.

Therefore, it is not reasonable to use generic injection boli for virtual angiography, but to gain this information from the current patient case instead. Furthermore, additional physical and physiologic effects cause the injection bolus to alter, which means that the bolus does not arrive at the aneurysm as set up originally (e.g., constant injection rate of 2 mL/s for 3 s, resulting in an ideal rectangular bolus). These effects are, for instance, caused by the inner resistance of the catheter [30] and the contrast agent diffusion, since the point of injection is usually located at a certain distance proximal to the aneurysm.

In order to consider these aspects for the virtual angiography, the injection bolus profile that we use in our approach is directly extracted from the acquired 2D DSA series. For this purpose, a time-intensity curve—as used for extracting heart rate and heart state information—is again employed, whose LOI is supposed to be located at the inlet plane of the CFD simulation, since that is where the virtual contrast agent is injected, as shown in Figure 5.

However, due to its mixing with blood, the contrast agent and hence the acquired time-intensity curve reflect the pulsatility caused by the patient's cardiac activity. Using this time-intensity curve directly as injection bolus profile would thus lead to the measured pulsatile pattern being included in the virtual angiography simulation. Since the velocity field generated by the CFD solver already implies a pulsatile pattern itself, this means that pulsatility impact would in fact be considered twice.

To eliminate this pulsatile pattern and further background noise, the measured data will be fit to a predefined function. In order to determine the best-fit model parameters, the Levenberg-Marquardt optimization algorithm is used [31], which iteratively solves least-square optimization problems for nonlinear functions using a combination of the steepest descent and the Gauss-Newton method.

For the sake of representing the profile of the bolus injection over time, an adapted function based on [30] is used. Analogous to the electrical behavior of a capacitor, this function is given by

**Figure 7:**
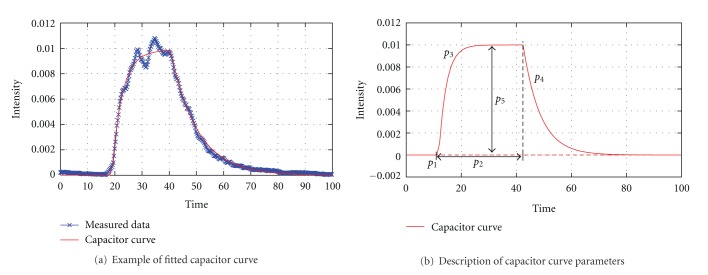
Capacitor function.

(11) I(p,t) ={t<p10,t≥p1∧t<p1+p2p5·(1−e−(t−p1)/p3),elsep5·(1−e−p1/p4)   ·(−e−(t−(p1+p2))/p4) ,

where the parameter set **p** = {*p*_*i*_},  *i* ∈ 1,…, 5 describes the curve according to Figure 7(b). To some extent, the curve resembles a rectangular function and hence rather originates from a constant contrast bolus injection which is just slightly altered by diffusive and inner resistance of the catheter. In Figure 7(a), an example of a capacitor curve fit is illustrated.

The fitted injection bolus curve *I*(*t*) is finally resampled at *n* points, where *n* denotes the number of time steps to simulate. By normalizing ∑_*i*=1_^*n*^ *I*_*i*_ = 1, a multiplication of *I*_*i*_ with the total number of particles to be injected results in the number of particles to be injected at time *i*.

### 2.4. 3D/2D Transformation—Forward Projection

Creating 2D projection images out of contrast agent concentration volumes can be described by a transformation *T* : ℝ^3^ → ℝ^2^, which corresponds to the X-ray acquisition in real angiographic procedures. For the virtual angiography, the simulated projection is supposed to be computed such that the viewing directions of real and virtual angiograms match.

X-ray imaging is based on a source emitting and an image detector collecting photons. Since the (idealized) X-ray source is a point source, this system can geometrically be described by a pinhole camera model based on perspective projections [32]. Within this model, the mapping *T* of a point **p** ∈ ℝ^3^, located between the source and the detector, onto the image plane can be expressed as a linear transformation (in case homogeneous coordinates are used [32]) and performed using matrix calculations. This allows to represent *T* by

(12) $$\begin{array}{c} p_{\text{proj}}^{\prime }=T\left(p\right)=P\cdot p\prime , \end{array}$$

where **p**′ ∈ ℝ^4^ is the homogeneous representation of the point **p**, *P* ∈ ℝ^3  ×  4^ is the projection matrix, and **p**_proj_′ is the projected point, given in homogeneous coordinates.

For the case of C-arm imaging in the angiography suite, projection matrices are used for several applications; for example, for the 3D image reconstruction process based on 3D RA acquisitions [33]. For that purpose, the individual positions of the C-arm during the acquisition are specified by the used protocol and hence known in advance, which allows to generate the projection matrices within a calibration run once when the system is installed or maintained [6]. However, the acquisition of 2D DSA series is in general performed using an arbitrary C-arm angulation, for which no calibrated projection matrix is available. Nevertheless, those projection matrices are needed for generating the virtual angiograms which correspond to the real ones concerning the viewing direction.

According to the study in [34], the projection matrices can generally be calculated based on available information of the C-arm system. For this purpose, the angulation of the C-arm in left/right (LAO/RAO) as well as in head/feet (CRAN/CAUD) direction, the pixel spacing of the detector, the source-image-distance (SID), the source-to-isocenter distance (SISOD), and the coordinates of the isocenter related to the image plane are required. This information can be retrieved from the DICOM header of the particular DSA series.

For these calculations, an idealized projection geometry of the system is supposed, which neglects for instance gantry motion as well as mechanical instabilities. This in general leads to projection matrices being less accurate than the calibrated ones, see [34] for details. The resulting virtual projection images may then slightly differ from the acquired images with respect to the viewing direction.

The calculated matrices are eventually used to project the contrast medium concentration volume onto the virtual image detector. Our framework uses an implementation based on the ray casting technique [35], which generates a ray for each pixel of the virtual image plane that intersects the corresponding pixel, the virtual X-ray source, and the concentration volume that is located between the virtual source and the virtual detector. The rays are then sampled equidistantly and the concentration values for these positions, which correspond to X-ray attenuation coefficients, are added up. This summation corresponds to the numerical approximation of the line integral of X-ray attenuation values along the respective ray.

### 2.5. Comparison/Evaluation—Methods

In total, three different cases were used for testing, each of them consisting of a 3D RA data set and at least one high-speed DSA series (30 fps) showing a complete bolus passage, see Table 1. For one case (patient A), two DSA series are available, which show the bolus injections from different C-arm angulations. The first case (phantom data) is a medical phantom of a giant artificial aneurysm, whose shape is based on a real patient case. The pulsatile blood flow is modeled using a combination of a steady and a pulsatile pump. The other cases (patient A, patient B) represent data from two patients with aneurysms at the internal carotid artery. The data was provided by Stony Brook University, New York (phantom data), and the Department of Neuroradiology, University of Erlangen-Nuremberg (patient A, patient B).

The evaluation is performed on a qualitative and a quantitative basis. For qualitative comparisons, features such as synchronization of time, a match of global flow patterns, zones of recirculation, and the location of the inflow jet are inspected.

For the comparison between virtual and real angiograms, a pixelwise correspondence is not given (see Section 2.4): Therefore, quantitative measurements are based on time-intensity curves (TICs) of certain regions of interest (ROI). These regions are selected by hand at corresponding positions of real and virtual angiograms.

**Figure 8:**
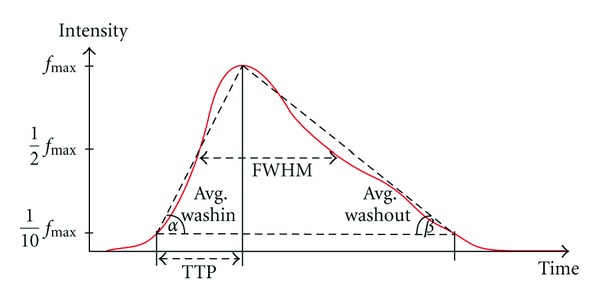
Illustration of quantitative features.

In detail, the quantitative features, as depicted in Figure 8, are as follows [17, 36]:

(i) Full width at half maximum (FWHM): this measurement describes the duration *d*_FWHM_ = *t*_2_ − *t*_1_ between two points in time where the measured intensities reach half of the maximum intensity during wash-in and wash-out phases; that is, *f*(*t*_1_) = *f*(*t*_2_) = (1/2)*f*_max⁡_. This parameter indicates the rate at which blood (or contrast agent) is exchanged in the selected region of interest and is practically used for evaluating the outcome of a treatment by comparing pre- and postmeasurements.
(ii) Time to peak (TTP): time to peak describes the duration *d*_TTP_ until the maximum opacification *f*_max⁡_, that is, intensity, is reached. The duration is measured from the time when the opacification reaches 10% of total opacification for the first time. This parameter quantifies the wash-in phase.
(iii) Average washin/washout: the average slope at which the time-intensity curve increases and decreases, respectively. For that purpose, the durations from 10% of maximum opacification until *f*_max⁡_ is reached and the decrease from *f*_max⁡_ to 10% of maximum opacification is used. The parameter is taken for describing the inflow and the outflow behavior, respectively.
(iv) Relative root-mean-square error (rRMSE, [17]) between time-intensity curves of real and virtual angiograms. The TIC corresponding to the real angiogram is scaled such that its values lie in the range between 0 and 100; the TIC of the virtual angiogram is then shifted and scaled such that the rRMSE measurement gets minimal. The rRMSE is defined as
  (13) $$\begin{array}{c} \text{rRMSE}=\sqrt{\frac{1}{n}\sum_{i=1}^{n}{\left(\frac{P_{i}-T_{i}}{T_{m}}\right)}^{2}}, \end{array}$$
  where *P*_*i*_ and *T*_*i*_ denote the (normalized) intensities of the TICs of real and virtual angiograms. *T*_*m*_ represents the mean value of the TIC of the acquired angiogram. For certain patient cases, venous structures may overlay the observed vascular segment in the background, leading to a rerise of intensities after a certain time. Since, for simulated cases, this situation does not occur, this measurement may be strongly corrupted by diverging time-intensity curves between real and virtual angiograms when the contrast agent reaches those venous structures. To avoid that problem, this curve-based measurement is restricted to a selected part of the time-intensity curve only instead of the total simulated duration.

## 3. Results and Discussion

### 3.1. Arbitrary Projection Angles

As was mentioned in Section 2.4, the used projection matrices are generated from C-arm angulation information in order to reproject the calculated contrast agent concentration volume according to the projection direction of the acquired 2D DSA sequence. This step is needed to compare the real and virtual angiograms side by side for the purpose of validating CFD simulation results.

**Figure 9:**
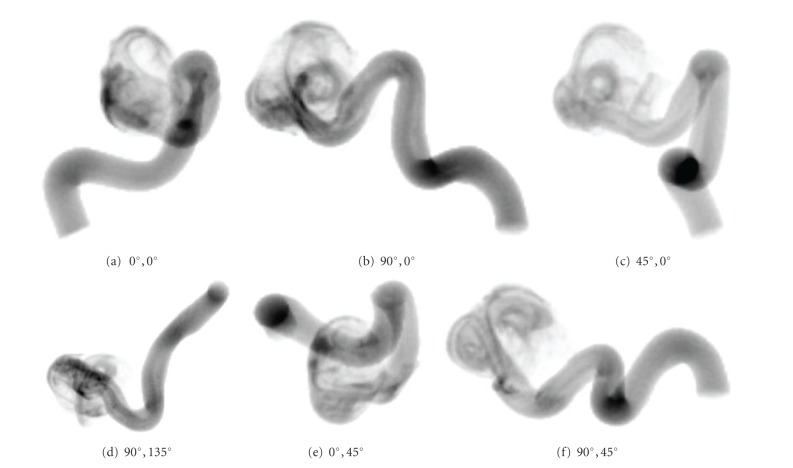
Virtual angiograms based on different projection angles for the same simulated angiography. All images show the contrast agent distribution at the same point in time. The simulated position of the C-arm is denoted below each image, given as a *primary angle*, *secondary angle* pair, where *primary angle* denotes the rotation about the left/right axis and *secondary angle* about the head/feet axis.

In principle, by choosing arbitrary values for the C-arm angulation (rotation in left/right and head/feet direction), arbitrary projection geometries are possible, compare Figure 9; this particularly enables angulations which cannot be reached in reality due to mechanical limitations.

It is important to note that the generation of additional virtual angiograms requires no further X-ray exposure or additional contrast agent delivery to the patient. Any number of virtual angiograms can be generated from any desired viewing angle without the need for additional imaging of the patient, thus potentially becoming an important tool in the treatment planning process for a number of cerebral vascular disorders.

### 3.2. Comparison of Real and Virtual Angiograms—Phantom Data

For the phantom data set, the simulated angiography was performed using a total of 10^6^ particles. *δt* was chosen to be 0.002 s, the diffusivity coefficient *D* = 0.1, and the smoothing parameter *σ* = 1.0. In total, 20.04 s were simulated, corresponding to the acquired DSA series (601 frames at 30 fps). The injection bolus was modeled using the presented capacitor function (Figure 7, (11)). From the acquired 2D DSA series, a heart rate of 84 bpm was extracted. The underlying CFD simulation was performed using a blood viscosity of 0.004 Pa·s with a density of 1050 kg/m^3^. The inflow profile was synchronized with the heart state as described, with velocities ranging from 0.28 to 0.37 m/s and an average of 0.31 m/s.

The real and the corresponding virtual angiogram is shown in Figure 11. Overall, the angiograms show satisfying accordance concerning the temporal synchronization and global flow patterns. In both angiograms, the inflow jet enters the aneurysm at *t* = 2.6 s and proceeds along the right aneurysm wall, as can be seen in Figures 11(a) and 11(b). Within the aneurysm dome, the contrast agent further circulates counter-clockwise Figures 11(c)–11(f). In Figures 11(c)–11(f), the real angiogram appears to have a slightly faster filling of the aneurym with contrast agent; having a closer look at the inflow jet, one can observe that the contrast agent for the real angiogram is distributed homogeneously very fast within the aneurysm dome, whereas the virtual contrast agent stays dense on the right side of the aneurysm Figure 11(d). Presumably, this different behavior might be a mismatch of assumed and real properties of blood, for instance viscosity, whose effects have not been observed in this study. For both cases, the opacification in the aneurysm reaches its maximum at approximately 4-5 s, compare Figures 11(g)-11(h). At *t* = 5.2 s (h), the inflow of contrast agent reduces and the remaining contrast agent is flushed out of the aneurysm Figures 11(i)–11(n). The outflow phase is well synchronized here. As can be seen in Figures 11(k)–11(n), a small amount of contrast agent remains at the bottom left side of the aneurysm in the real angiogram.

**Figure 10:**
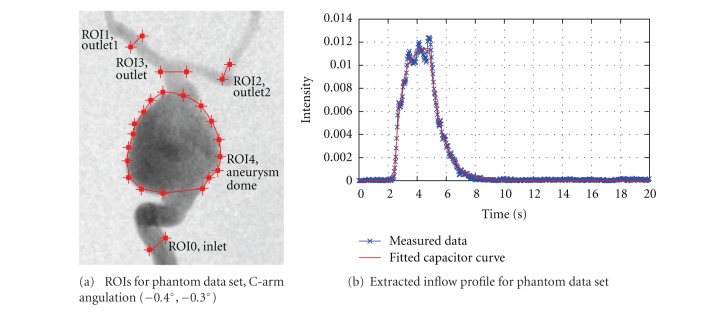
ROIs of phantom data set and used inflow profile.

For this case, multiple regions of interest for measuring time-intensity curves are chosen, as depicted in Figure 10(a), to measure certain effects. ROI0, which is placed at a short distance behind the particle injection area, is supposed to show that certain conditions (heart rate, heart state, contrast bolus, synchronization of time) are in agreement for the beginning of the simulated and the real domain. ROI1, ROI2, and ROI3 measure the contrast bolus at the end of the simulated domain. Assuming identical measurements between real and virtual angiograms at ROI0, differences which occur in one of these ROIs might indicate a different behavior of contrast agent for the real and simulated environment for preceding areas. Finally, ROI4 is chosen to cover the whole aneurysm dome to measure the global behavior of contrast agent within that domain.

In Figure 12, the measured time-intensity curves for the regions of interest depicted in Figure 10(a) are shown. The curves are normalized such that the measured intensities range from 0 to 100 for both angiograms. For the real angiogram, noise and motion cause the time-intensity curves to appear more ragged.

To demonstrate the importance of extracting the injection-specific bolus profile and the synchronization of the virtual contrast agent injection with the patient's cardiac activity according to the presented methods, time-intensity curves are additionally presented for a virtual angiography based on a generic, rectangular injection bolus. For that case, the duration of the injection is adapted to the FWHM measurement of a time-intensity curve acquired at the LOI shown in Figure 5(b).

In Figure 12(a), the time-intensity curves are given for the region of interest located at a short distance behind the particle injection area (ROI0). The curves are well synchronized in shape and time, which indicates a proper extraction of the contrast bolus injection profile, see Figure 10(b). The pulsatility in both angiograms further coincides well concerning the duration of the cardiac cycle and the synchronization of the heart phase, as can be seen in box 1, Figure 12(a). Differences in the height of the intensity peaks are possibly caused by the inflow velocities which are used for the CFD simulation. For this case, the range of presumed inflow velocities (0.28–0.37 m/s) is propably chosen too wide which may cause the intensities of the virtual angiogram to oscillate stronger than those of the real angiogram.

The time-intensity curves for the outlets, that is, ROI1, Figure 12(b) and ROI2, Figure 12(c), show a strong correspondence for the rising edge between real and virtual angiograms, meaning that contrast agent arrives synchronously in time within the real and virtual angiogram.

**Figure 11:**
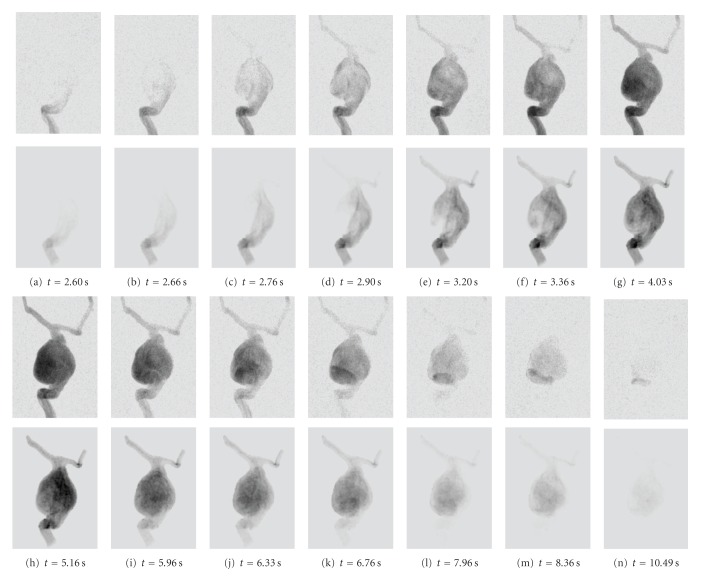
Real (1st and 3rd row) and virtual (2nd and 4th row) angiograms of phantom data for different time steps, which are denoted below the images. *t* = 0 s corresponds to the respective beginning of the DSA sequence.

**Figure 12:**
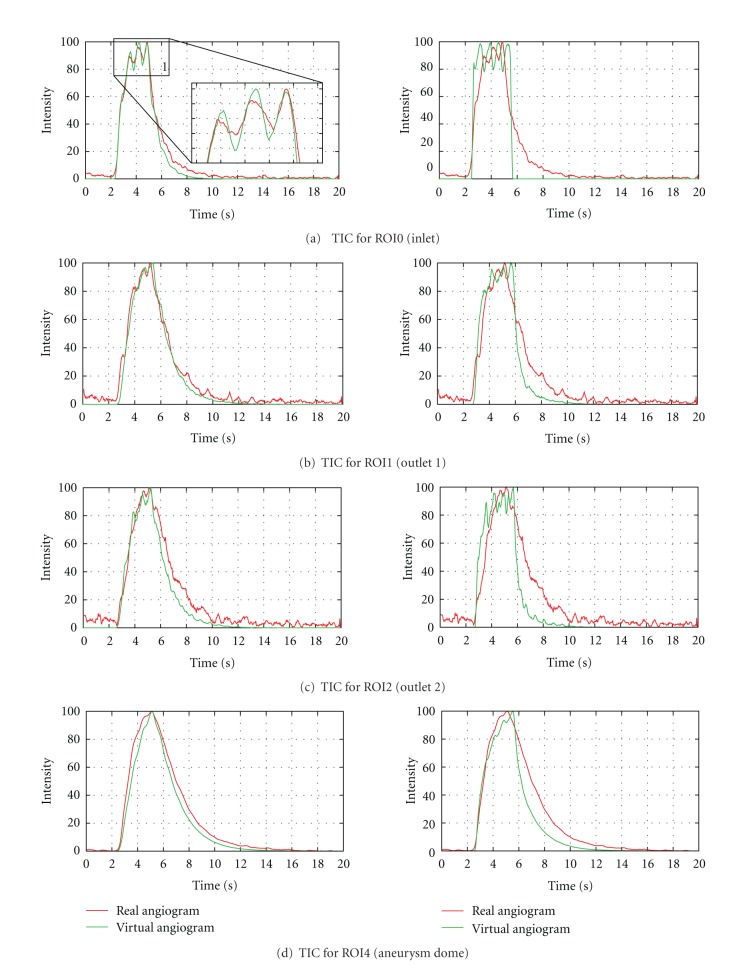
Time-intensity curves for phantom data set, based on real and virtual angiograms. On the left side, the heart state is synchronized and an injection bolus based on a capacitor function is used, whereas on the right side no synchronization is performed and a rectangular bolus profile is used.

In Figure 12(d), the time-intensity curves are compared for the region of interest covering the complete aneurysm dome. According to this figure, the maximum opacification is reached for both angiograms identically after 5 s, which has also been observered in Figure 11.

Table 2 denotes the quantitative values for the measured time-intensity curves. The measured FWHM is slightly increased (0.53 s) for the real aneurysm dome in contrast to that from the virtual angiogram. The FWHM at ROI0 (inlet) is in good agreement (difference: 0.07 s), but for the successive regions (outlet ROIs), an increased gap is detected (differences: 0.33 s–0.39 s).

The time to peak measurements coincide for ROI1–ROI4; the large difference for the inlet ROI (ROI0) is caused by the fact that the maximum opacification of the real curve is reached at the third main intensity peak, while the one of the virtual angiogram is already reached at the second peak, see box 1 in Figure 12(a).

For the washin phase, the values correspond to a great extent; the average washin for ROI0, which is increased for the real angiogram, arises from the peak-to-peak shift described above. Concerning the washout phase, the real angiogram has a decreased average washout rate (ROI1–ROI4), which might be caused by the remaining contrast agent within the aneurysm dome.

**Figure 13:**
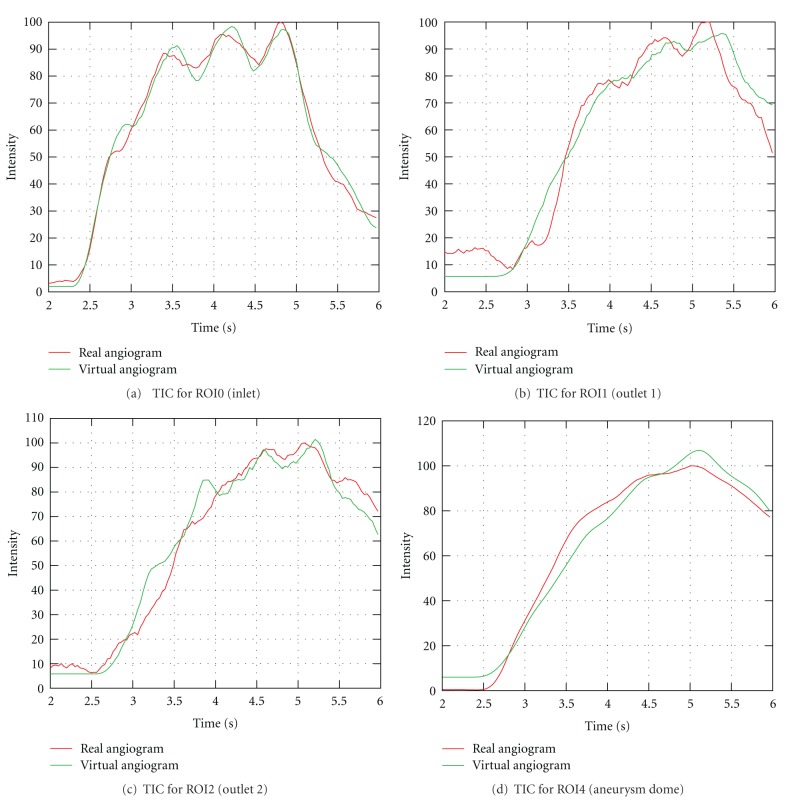
Time-intensity curves for phantom data set, based on real and virtual angiograms. The curves are cropped to arterial phase.

The calculated relative root mean square errors for the arterial phase of the time-intensity curves, see Figure 13, are in the range of 5.3%–13.3% and thus comparable to [17].

### 3.3. Comparison of Real and Virtual Angiograms—Patient A

For patient A, who has a large/giant aneurysm at the internal carotid artery, two angiograms with C-arm angulations, (0°, 0°; anteroposterior view) and (−91°,−0.2°; lateral view), were evaluated. The parameter set is chosen for both cases as follows: 10^6^ particles were used, *δt* was chosen to be 0.002 s, diffusivity *D* = 1, and smoothing factor *σ* = 1. The acquired DSA sequence has a duration of 12.4 s. The extracted heart rate is 81 bpm. The CFD inflow velocities have a mean of 0.31 m/s, ranging from 0.25 to 0.42 m/s. Blood specific parameters were chosen identical as for the phantom data.

#### 3.3.1. Projection 1 (0° RAO/LAO, 0° CRAN/CAUD)

**Figure 14:**
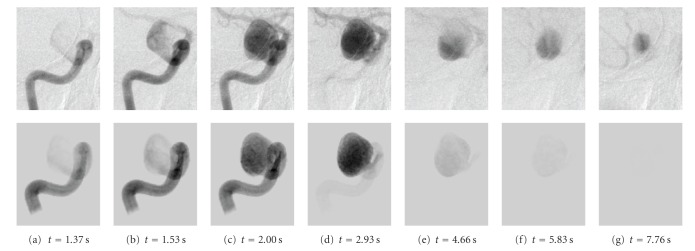
Real (1st row) and virtual (2nd row) angiograms of patient A, projection 1, for different time steps, which are denoted below the images. *t* = 0 s corresponds to the beginning of the DSA sequence.

Figure 14 shows both angiograms. The inflow of contrast agent is well synchronized between the real and virtual angiogram, Figures 14(a) and 14(b). The inflow jet, which is clearly visible in both cases, pours into the aneurym at the bottom side and circulates clockwise. The depicted part of the vessel is homogeneously filled with contrast agent in (c). At *t* = 2.93 s, the inflow phase is finished for both angiograms.

Major differences between the real and virtual angiogram occur during the washout phase. In the virtual angiogram, the contrast agent is flushed out quite fast, whereas in the real angiogram, a remaining part of contrast agent still stays inside the aneurysm Figures 14(e)–14(g). 

**Figure 15:**
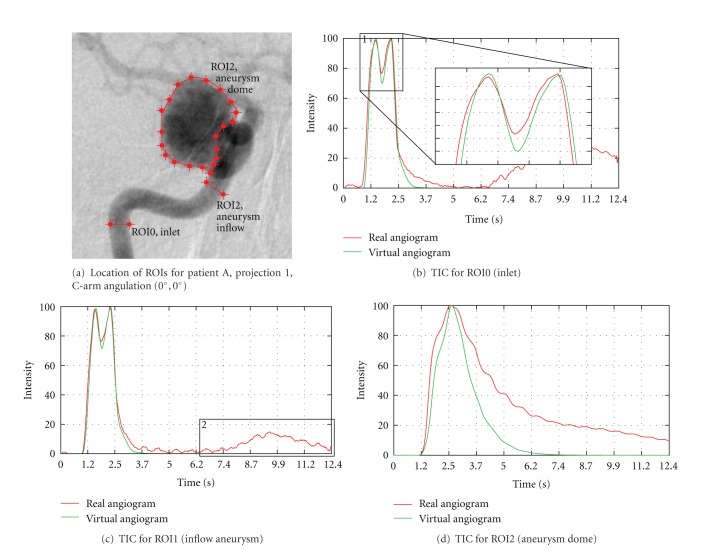
Time-intensity curves for patient A, projection 1, based on real and virtual angiograms.

Identical characteristics can be observed for the time-intensity curves in Figure 15 and the measured quantities based on these curves, as denoted in Table 3. The synchronization (time, heart rate, and heart state) for the inlet (ROI0) is given to a great extent, compare Figure 15(b). The full width at half maximum for that measurement differs only by 0.04 s or approximately by one image frame. The deviation concerning the averaged washin and washout can be explained by looking at the zoomed section, see box 1 in Figure 15(b). Both curves have two intensity peaks; for the real angiograms, the measured intensity is larger at the second peak, while the virtual angiogram reaches its maximum at the first peak. This deviation is 0.69 s, which causes the mentioned difference.

An additional effect, which can usually be avoided for medical phantoms, but which is a common observation for actual patient cases is the depicted rerise of intensity values towards the end of the sequence, see TICs in Figures 15(b) and 15(c), box 2. This effect is caused by contrast agent in arteries and veins which are in the background of the observed aneurysm, but do overlay with the aneurysm on the 2D DSA images and hence contribute to the opacification.

**Figure 16:**
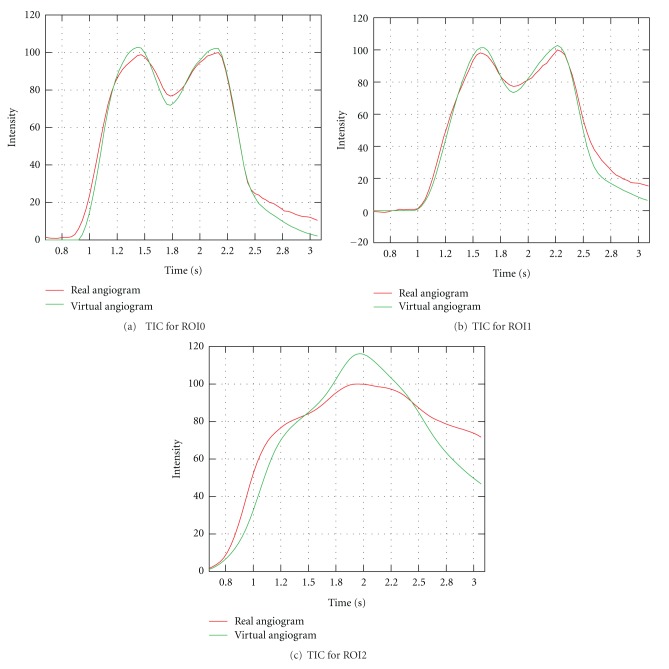
Time-intensity curves for patient A, projection 1, based on real and virtual angiograms. The curves are cropped to arterial phase.

The rRMSE, which is calculated from the time-intensity curves cropped to arterial phase (Figure 16), is comparable for ROI0 and ROI1. For ROI2, the rRMSE is increased due to the observed remaining contrast agent inside the aneurysm.

#### 3.3.2. Projection 2 (−91° RAO/LAO, −0.2° CRAN/CAUD)

**Figure 17:**
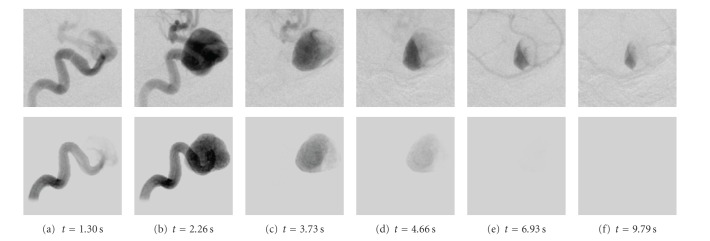
Real (1st row) and virtual (2nd row) angiograms of patient A, projection 2, for different time steps, which are denoted below the images. *t* = 0 s again corresponds to the beginning of the DSA sequence.

The second projection provides a lateral view (rotated by 90° in left/right direction compared to projection 1) of the aneurysm, see Figure 17. After the washin phase, the contrast agent circulates in the left part of the aneurysm, both in the real and virtual angiograms (c). As was observed in the first projection, the contrast agent flushes out quite fast in the virtual angiogram, whereas in the real angiogram, a small portion remains within the aneurym, Figures 17(d)–17(f). From this angulation, it is clearly visible that the contrast agent is settling at the left side of the aneurysm (direction of gravity). This effect, although not as intense, has also been observed for the acquired angiogram of the phantom data, compare Figures 11(k)–11(n). This settling of contrast agent is a known physiological effect [37–39], but the reason for that behavior has not finally been discovered yet.

**Figure 18:**
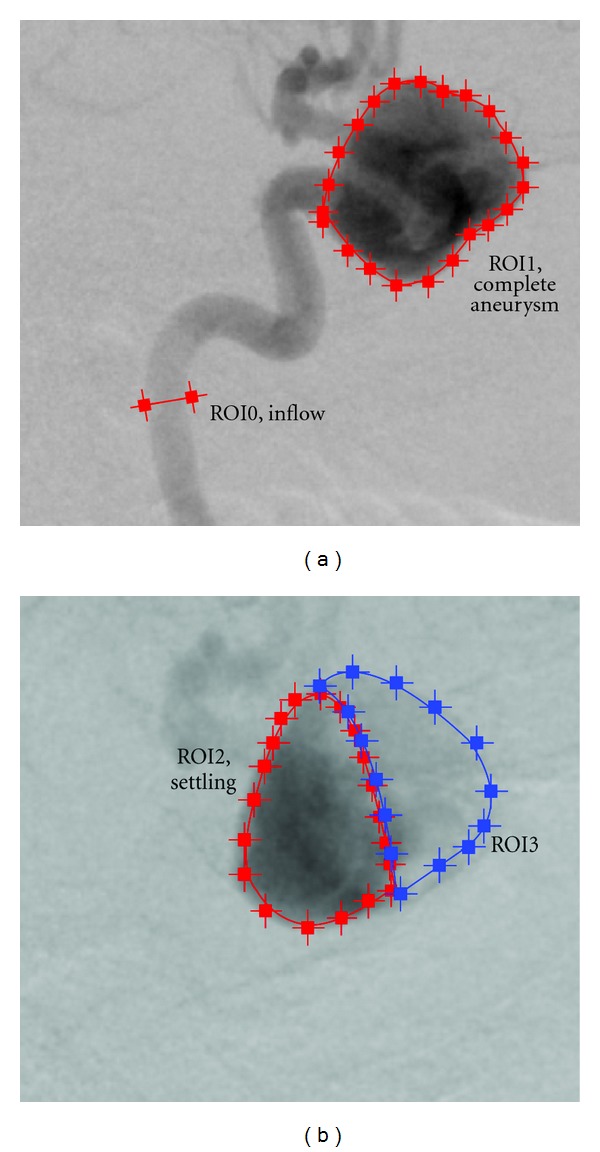
Location of ROIs for patient A, projection 2, C-arm angulation (−91°, −0.2°).

**Figure 19:**
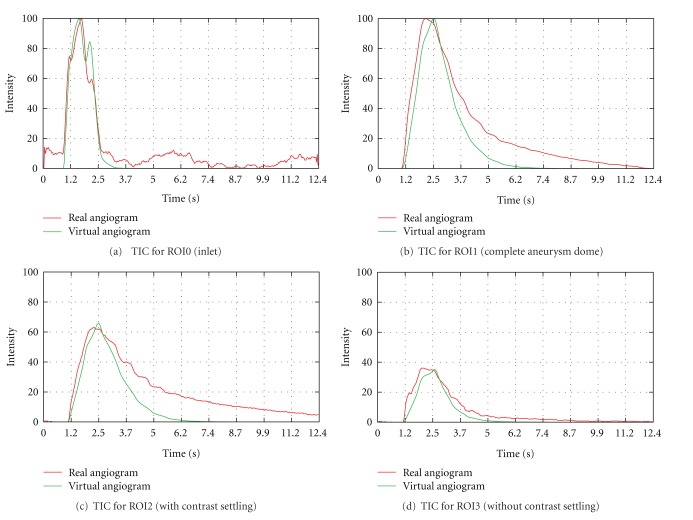
Time-intensity curves for patient A, projection 2, based on real and virtual angiograms.

For taking a closer look at this effect, we measured time-intensity curves for the regions of interest depicted in Figure 18 and derived quantitative values for these regions, see Table 4. For the complete aneurysm (ROI1), the time-intensity curve of the real angiogram, Figure 19(b), has a sustained wash-out phase, similar to the corresponding curve of the previous projection, compare Figure 15(d).

**Figure 20:**
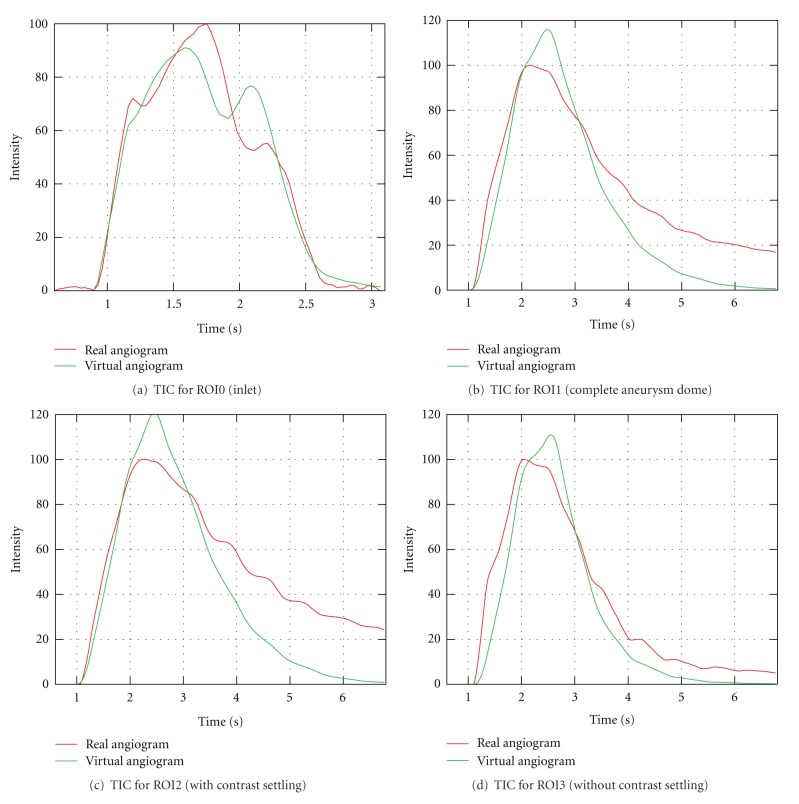
Time-intensity curves for patient A, projection 2, based on real and virtual angiograms. The curves are cropped to arterial phase.

In Figures 19(c) and 19(d), this settling effect is further investigated. The region of interest covering the complete aneurysm dome (ROI1) is partitioned into two distinct regions capturing the areas with (ROI2) and without (ROI3) settled contrast agent. This partitioning is illustrated in Figure 18. The corresponding time-intensity curves, as depicted in Figures 19(c) and 19(d), are normalized with respect to the normalization factors of the time-intensity curves in Figure 19(b), meaning that the addition of the corresponding curves in Figures 19(c) and 19(d) results in the curves of Figure 19(b). As can been seen in the two figures, we were able to separate the settling effect. The trailing edge of the real curve in Figure 19(b) is almost identical to the trailing edge for the curve in Figure 19(c) in its shape and height. In comparison, the real curve in Figure 19(d) has no extended outflow phase and fits quite well to the virtual curve. The TICs cropped to arterial phase, which are used for calculating the rRMSE, are provided in Figure 20.

Consequently, the principle behavior of contrast agent within the aneurysm is captured sufficiently. However, additional effects such as this mixing/settling behaviour occur in real environments and are not covered by our model yet.

### 3.4. Comparison of Real and Virtual Angiograms—Patient B

Patient B has a medium-sized aneurysm at the supraophthalmic internal carotid artery. One acquired angiogram is available (C-arm angulation 16.4°, 16.1°), which has a duration of 6.7 s. Contrast agent was injected for only a small time period, resulting in a short measured injection bolus. For the region of interest at a short distance behind the inlet (ROI0, Figure 22), the FWHM of the measured time-intensity curve is 0.59 s, as given in Table 5—there was no possibility to extract information about heart rate and heart state, see Figure 23. Therefore, a heart rate of 69 bpm was assumed, and the CFD velocity inflow profile could not be synchronized with the acquired angiogram. The selected inflow velocities range from 0.44 to 0.6 m/s, the average velocity is 0.5 m/s. For the simulation, the parameters were chosen as for the other patient case (10^6^ particles, *σ* = 1, *δt* = 0.002 s), with a diffusivity coefficient *D* = 1.0.

**Figure 21:**
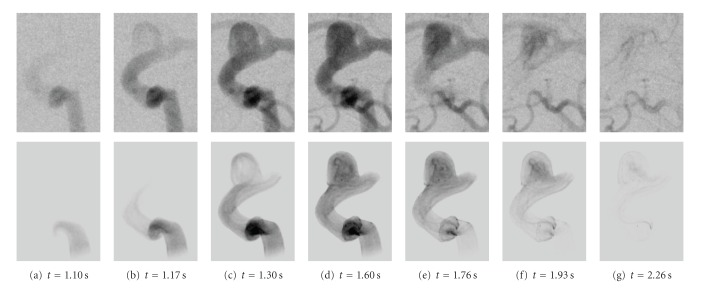
Real (1st row) and virtual (2nd row) angiogram of patient B for different time steps, which are denoted below the images. *t* = 0 s corresponds to the beginning of the DSA sequence.

**Figure 22:**
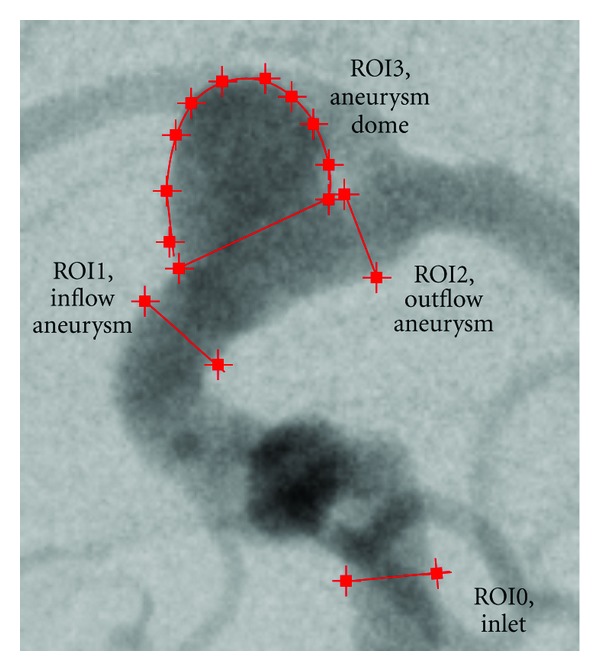
ROIs for patient B.

Figure 21 shows the real and virtual angiograms. The global flow patterns of both angiograms match, as can be seen from the inflow jet entering the aneurysm, compare Figure 21(b) for the real and Figure 21(c) for the virtual angiogram, and the small amount of contrast agent in the center of the aneurysm before it is washed out, see Figure 21(f). Figures 21(a)–21(c) reveal that the filling of the parent vessel and of the aneurysm itself is delayed for the virtual angiogram compared to the real angiogram, which presumably indicates an underestimation of inflow velocities for the CFD simulation.

**Figure 23:**
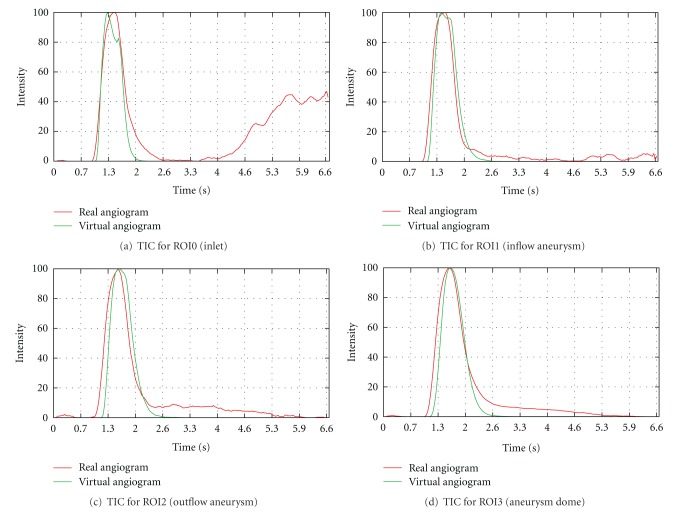
Time-intensity curves for patient B, based on real and virtual angiograms.

This observation can also be retrieved from the time-intensity curves. From ROI0 to ROI2, Figures 23(a)–23(c), a shift between both curves is observable, which is progressively growing with increasing distance from the injection spot. The intensities of the real angiogram are thereby registered sooner than those of the virtual angiogram.

**Figure 24:**
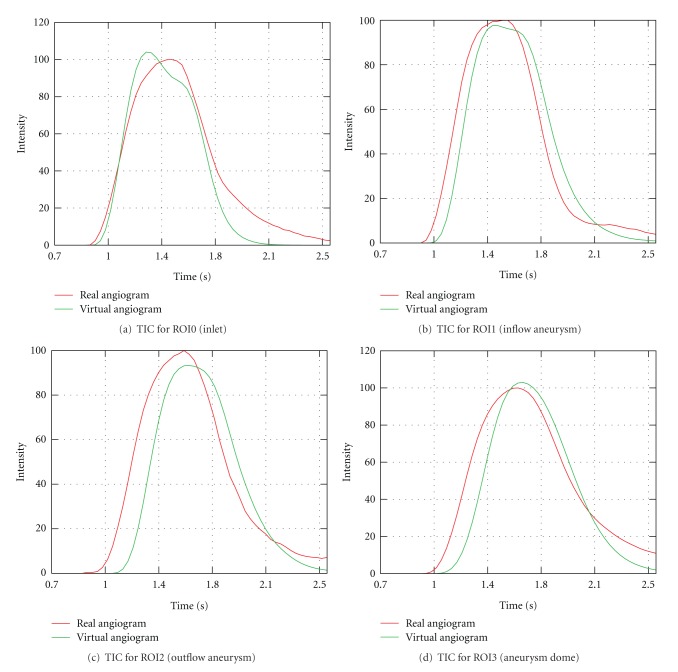
Time-intensity curves for patient B, based on real and virtual angiograms. The curves are cropped to arterial phase.

In general, further calculated quantities based on time-intensity curves cropped to arterial phase, Figure 24, show a concordance between real and virtual angiograms, meaning that the overall simulated behavior matches satisfactorily the *in vivo* behavior, see Table 5. However, the delay strongly affects the relative root mean square error, which is significantly larger than for the previous cases under consideration.

## 4. Conclusion

In this paper, we have proposed a workflow to generate patient-specific virtual angiograms based on CFD simulation results.

We have used discrete particles in order to simulate the transport of contrast agent and successfully demonstrated that this approach can properly model the behavior of contrast agent, although this is not the predominant approach used in related works [10, 30].

As a particular aspect, we have put particular emphasis on a patient-individualized generation of virtual angiograms, namely, the incorporation of the patient-individual heart rate as well as the treatment-specific bolus injection profile caused by manual contrast medium injection, for example. As a consequence of this approach, it is now possible to apply our virtual angiography method without the need of using contrast injection systems or taking care of specified contrast bolus profiles, which may improve the future usability of this application in clinical settings.

To obtain these parameters, we presented methods which solely used the acquired 2D DSA data. Satisfying results have been achieved with these methods to synchronize the heart rate, heart state, and contrast bolus injection of virtual and acquired angiograms. In general, the measured quantitative differences are approximately in the same range between real and virtual angiograms as given in [17]. Further improvements of the results may be possible due to more sophisticated methods for adapting the inflow velocities.

Finally, the overall correspondence offers the possibility to use the presented virtual angiography workflow as a tool towards the indirect validation of patient-individual CFD simulation results.

## Figures and Tables

**Table 1:** Cases used for testing and evaluation.

Data set	3D RA data		2D DSA	
	Size (voxel)	Resolution (mm)	Resolution (mm)	Frame rate
Phantom data	512 × 512 × 512	0.46 × 0.46 × 0.46	0.308 × 0.308	30 fps
Patient A	512 × 512 × 396	0.28 × 0.28 × 0.28	0.308 × 0.308	30 fps
Patient B	512 × 512 × 396	0.1 × 0.1 × 0.1	0.154 × 0.154	30 fps

**Table 2:** Quantitative measurements for phantom data.

DSA	ROI	FWHM	⌀ Washin (rad)	⌀ Washout (rad)	TTP	rRMSE
Real	ROI0,	2.74 s	0.89	−0.84	2.54 s	5.3%
Virtual	Inlet	2.67 s	1.05	−0.89	1.82 s	

Real	ROI1,	3.17 s	0.89	−0.66	2.51 s	13.3%

Virtual	Outlet1	2.84 s	0.89	−0.79	2.51 s

Real	ROI2,	3.10 s	0.90	−0.59	2.44 s	10.8%

Virtual	Outlet2	2.71 s	0.91	−0.80	2.44 s

Real	ROI3,	3.17 s	0.87	−0.61	2.67 s	7.6%

Virtual	Outlet	2.81 s	0.90	−0.80	2.44 s

Real	ROI4,	3.73 s	0.90	−0.55	2.48 s	9.3%
Virtual	Aneurysm dome	3.20 s	0.91	−0.62	2.41 s	

**Table 3:** Quantitative measurements for patient A, projection 1.

DSA	ROI	FWHM	⌀ Washin (rad)	⌀ Washout (rad)	TTP	rRMSE
Real	ROI0,	1.29 s	1.18	−1.28	1.29 s	9.2%

Virtual	Inlet	1.25 s	1.42	−1.16	0.5 s

Real	ROI1,	1.29 s	1.20	−1.29	1.22 s	9.9%

Virtual	Aneurysm inflow	1.22 s	1.20	−1.34	1.22 s

Real	ROI2,	2.87 s	1.20	−0.29	1.22 s	16.2%
Virtual	Aneurysm dome	1.72 s	1.20	−0.93	1.22 s	

**Table 4:** Quantitative measurements for patient A, projection 2.

DSA	ROI	FWHM	⌀ Washin (rad)	⌀ Washout (rad)	TTP	rRMSE
Real Virtual	ROI0,	1.29 s	1.29	−1.20	1.29 s	23.4%
	Inlet	1.25 s	1.37	−1.24	0.5 s	

Real Virtual	ROI1,	2.21 s	1.25	−0.50	1.22 s	33.6%
	Complete aneurysm	1.65 s	1.18	−0.92	1.22 s	

Real Virtual	ROI2,	2.61 s	1.23	−0.44	1.22 s	38.0%
	aneurysm part w/settling	1.78 s	1.18	−0.88	1.22 s	

Real Virtual	ROI3,	1.95 s	1.28	−0.78	1.22 s	30.3%
	aneurysm part w/o settling	1.45 s	1.18	−1.08	1.22 s	

**Table 5:** Quantitative measurements for patient B.

DSA	ROI	FWHM	⌀ Washin (rad)	⌀ Washout (rad)	TTP	rRMSE
Real	ROI0,	0.59 s	1.42	−1.31	0.5 s	35.2%
Virtual	Inlet	0.56 s	1.48	−1.39	0.3 s	

Real	ROI1,	0.63 s	1.41	−1.29	0.53 s	42.8%
Virtual	Aneurysm inflow	0.59 s	1.44	−1.37	0.43 s	

Real	ROI2,	0.66 s	1.41	−1.32	0.53 s	54.6%
Virtual	Aneurysm outflow	0.63 s	1.42	−1.37	0.50 s	

Real	ROI2,	0.73 s	1.41	−1.25	0.53 s	27.9%
Virtual	Aneurysm dome	0.63 s	1.43	−1.36	0.46 s	
